# Circulating tumor plasma cells correlate with tumor burden and routine laboratory markers in newly diagnosed multiple myeloma: A retrospective single-center study

**DOI:** 10.1097/MD.0000000000051073

**Published:** 2026-10-16

**Authors:** Xiaolei Du, Di Hu, Wenqiang Fu, Henggui Hu, Wenwen Chen, Xiaomeng Xu, Yuehong Li, Heng Tang, Mengyu Zhang

**Affiliations:** a Wanbei Coal Electric Group General Hospital, Suzhou, Anhui, China.

**Keywords:** circulating tumor plasma cells, flow cytometry, immunophenotype, multiple myeloma, tumor burden

## Abstract

Circulating tumor plasma cells (CTPCs) detected by multiparameter flow cytometry may serve as a minimally invasive marker of disease burden in multiple myeloma (MM). However, the relationship between CTPC burden, routine laboratory parameters, and bone marrow (BM) involvement remains insufficiently characterized in regional clinical settings. This retrospective single-center study included 51 consecutive patients with newly diagnosed MM. Peripheral blood CTPCs were quantified using ten-color flow cytometry. Among them, 41 patients with evaluable CTPC data were stratified into CTPC-higher (n = 20) and CTPC-lower (n = 21) groups using a 0.07% cutoff. Laboratory variables and immunophenotypic features were compared between groups, and correlations were assessed using Spearman rank analysis. Exploratory logistic regression adjusted for age and sex was performed, and Benjamini–Hochberg false-discovery-rate correction was applied to multiple comparisons. Compared with the CTPC-lower group, the CTPC-higher group had lower hemoglobin levels (73.5 vs 107.0 g/L; *P* < .001), lower albumin levels (30.9 vs 38.2 g/L; *P* = .006), higher β2-microglobulin levels (6.97 vs 3.25 mg/L; *P* = .004), and higher BM plasma cell percentages on smear examination (34.75% vs 12.00%; *P* = .005). CTPC levels were positively correlated with BM plasma cell percentage on smear examination (ρ = 0.67; *P* < .001) and negatively correlated with hemoglobin levels (ρ=−0.68; *P* < .001). These group differences and correlations remained significant after false-discovery-rate correction (all *q* ≤ 0.018). In age- and sex-adjusted analyses, hemoglobin, albumin, β2-microglobulin, and BM plasma cell percentage on smear remained associated with CTPC-higher status. Higher peripheral blood CTPC levels were associated with greater BM tumor burden and more abnormal routine laboratory markers in patients with newly diagnosed MM. CTPC quantification may serve as an adjunctive marker for baseline disease assessment in regional clinical settings. These cross-sectional findings from this limited sample do not establish prognostic value.

## 1. Introduction

Multiple myeloma (MM) is a clonal plasma cell malignancy characterized by bone marrow (BM) infiltration and monoclonal protein production. Patients commonly present with anemia, renal impairment, hypercalcemia, osteolytic bone disease, infection, or other nonspecific symptoms. Diagnosis and baseline assessments are based on BM evaluation together with laboratory and clinical findings.^[1,2]^ Although BM aspiration and biopsy remain essential in routine practice, marrow-based assessment is invasive and may be influenced by uneven distribution of plasma cell infiltration. Recent studies have shown that MM is spatially heterogeneous, supporting the need for complementary markers that can reflect the systemic disease burden.^[3]^

Circulating tumor plasma cells (CTPCs) are clonal plasma cells detected in the peripheral blood. Their presence suggests tumor cell dissemination beyond the BM microenvironment, and has been associated with more aggressive disease features in MM. Multiparameter flow cytometry enables the quantitative detection of CTPCs at low levels and has been increasingly used for the assessment of newly diagnosed MM.^[4,5]^ In a large prospective study of transplant-eligible patients with newly diagnosed MM, a CTPC cutoff of 0.07% was reported to identify patients with more aggressive disease and inferior outcomes.^[6]^ However, most available data are generated from clinical trial populations or large referral centers, and evidence from regional hospitals remains limited.

In regional clinical settings, clinicians often rely on routine laboratory markers, including hemoglobin, albumin, β2-microglobulin (β2-MG), lactate dehydrogenase (LDH), creatinine, calcium, and BM plasma cell percentage, to estimate the baseline disease burden. Clarifying the relationship between CTPC levels and these conventional parameters may help clinicians interpret the CTPC results in a practical context. Additionally, flow cytometry provides immunophenotypic information on clonal plasma cells, which may further characterize disease-related biological features.^[7,8]^

Therefore, this retrospective single-center study evaluated the peripheral blood CTPCs in patients with newly diagnosed MM. Using the previously reported 0.07% cutoff as an analytical grouping threshold,^[6]^ we investigated the association of CTPC levels with BM plasma cell burden, routine laboratory markers, and immunophenotypic features. As this study was cross-sectional and did not include survival follow-up, this cutoff was used only for baseline stratification, and no prognostic validation was attempted. The practical contribution of this study was the integrated assessment of CTPC burden alongside routinely available laboratory and BM measures in a regional real-world cohort.

## 2. Methods

### 2.1. Study population

This retrospective single-center study included consecutive patients with newly diagnosed MM admitted to the Wanbei Coal Electric Group General Hospital between January 2023 and December 2024. All diagnoses were established according to the International Myeloma Working Group criteria.^[1]^

A total of 51 patients were initially included, all of whom had complete BM immunophenotypic records. Among them, 41 patients had evaluable peripheral blood CTPC data and were included in the CTPC-specific group comparisons and correlation analyses. The remaining 10 patients did not undergo peripheral blood flow cytometry and were therefore excluded from the analyses involving CTPC levels. This study was approved by the Ethics Committee of Wanbei Coal Electric Group General Hospital (WBZY-LLWYH-2026-005) in accordance with the Declaration of Helsinki. As this study was retrospective, non-interventional, and based on anonymized clinical records, the requirement for written informed consent was waived by the ethics committee.

### 2.2. Flow cytometric detection of CTPCs and immunophenotyping

Peripheral blood and BM specimens were evaluated by multiparameter flow cytometry on a 3-laser, 10-color NAVIOS flow cytometer (Beckman Coulter). To reduce interference from plasma immunoglobulins (Igs), samples were washed before staining. The antibody panel included cKappa-FITC, cLambda-PE, CD27-ECD, CD117-PC5.5, CD19-PC7, CD138-APC, CD56-A700, CD38-A750, CD81-PB, and CD45-KrO antibodies. Here, cKappa and cLambda denote cytoplasmic kappa and lambda light chains, respectively.

The cell suspensions were adjusted to approximately 1 × 10^6^ cells/mL and incubated with surface monoclonal antibodies for 15 minutes in the dark. After erythrocyte lysis and washing, the cells were fixed and permeabilized using IntraPrep Reagent (Beckman Coulter) for intracellular light chain staining. Data acquisition targeted 100,000 to 500,000 nucleated events per sample, depending on specimen cellularity, with acquisition continued toward the upper end of this range when technically feasible. Instrument performance was verified before acquisition using routine manufacturer-recommended quality-control procedures, and the same instrument, antibody panel, acquisition settings, and compensation framework were used throughout the study.

Plasma cells were identified using a CD45/CD38/CD138-based gating strategy. Aberrant clonal populations were distinguished from residual normal plasma cells by their overall aberrant immunophenotypic pattern, supported by cytoplasmic light-chain restriction when present. The CTPC burden was calculated as the proportion of clonal plasma cells among the total nucleated cells in the peripheral blood. Antigen positivity was defined as expression in at least 20% of the aberrant clonal plasma cells. Positive and dim categories were assigned relative to residual normal plasma cells and internal negative populations; dim expression was retained separately and included in the reported combined positive/dim rate. The median fluorescence intensity (MFI) was recorded for the quantitative analysis of antigen expression. MFI values were reported in raw instrument units and were not converted to standardized fluorescence units. Peripheral blood data were used for CTPC quantification, whereas the antigen positivity and MFI results in Tables 2 and 3 were based on aberrant BM plasma cells. All flow cytometric data were analyzed using Kaluza software (Beckman Coulter). Representative BM immunophenotypic plots are shown in Figure 1. The assay was a local routine clinical protocol rather than a formally validated EuroFlow next-generation-flow assay, and no study-specific lower limit of detection or lower limit of quantification was established. No separate research-specific analytical reproducibility study was performed.

**Table 2:** Bone marrow plasma-cell immunophenotypic positivity in newly diagnosed multiple myeloma.

Antigen	Positive, n (%)	Dim, n (%)	Negative, n (%)	Combined positive/dim rate (%)
CD138	51 (100.0)	0 (0.0)	0 (0.0)	100.0
CD38	37 (72.5)	13 (25.5)	1 (2.0)	98.0
CD45	6 (11.8)	6 (11.8)	39 (76.5)	23.5
CD19	1 (2.0)	0 (0.0)	50 (98.0)	2.0
CD56	46 (90.2)	5 (9.8)	0 (0.0)	100.0
CD27	20 (39.2)	2 (3.9)	29 (56.9)	43.1
CD81	3 (5.9)	0 (0.0)	48 (94.1)	5.9
CD117	28 (54.9)	1 (2.0)	22 (43.1)	56.9

n = 51. Dim expression was retained as a separate category and included with unequivocally positive expression in the reported combined positive/dim rate. For CD56, 46 cases were positive and 5 were dim; no CD81-dim cases were observed. Monoclonal light-chain restriction was observed in 50/51 cases (kappa-restricted, 29; lambda-restricted, 21). Antigen positivity was defined as expression in ≥20% of aberrant clonal plasma cells.

**Table 3:** Quantitative surface-antigen expression (MFI) and comparison between CTPC groups.

Marker (MFI)	All (n = 51)	CTPC-higher (n = 20)	CTPC-lower (n = 21)	*P* value	FDR-adjusted *q* value
CD138	115.44 (84.12–216.64)	100.02 (75.36–129.32)	176.75 (92.24–230.01)	.043	0.209
CD38	115.74 (65.19–143.67)	110.54 (60.78–136.43)	121.92 (65.18–148.96)	.948	0.948
CD19	1.21 (0.76–2.06)	1.15 (0.79–1.75)	1.73 (0.94–3.11)	.068	0.209
CD56	81.40 (33.62–142.75)	60.62 (10.51–129.35)	111.82 (64.76–175.44)	.121	0.214
CD45	16.88 (6.29–29.90)	13.77 (5.21–18.42)	19.91 (4.94–30.07)	.531	0.607
CD27	5.81 (4.40–8.98)	5.23 (4.15–6.10)	6.58 (5.07–9.04)	.179	0.239
CD81	1.95 (0.98–2.84)	1.48 (0.78–2.30)	1.96 (1.15–3.09)	.134	0.214
CD117	4.81 (2.92–8.25)	3.99 (3.00–5.67)	7.64 (4.37–11.55)	.078	0.209

Groups were compared using the Mann–Whitney *U* test. *q* values were obtained using the Benjamini–Hochberg false-discovery-rate procedure across the 8 surface-antigen comparisons. MFI values are raw instrument units and were not calibrated to standardized fluorescence units.

CTPC = circulating tumor plasma cell, FDR = false discovery rate, MFI = median fluorescence intensity, presented as median (interquartile range).

**Figure 1.**
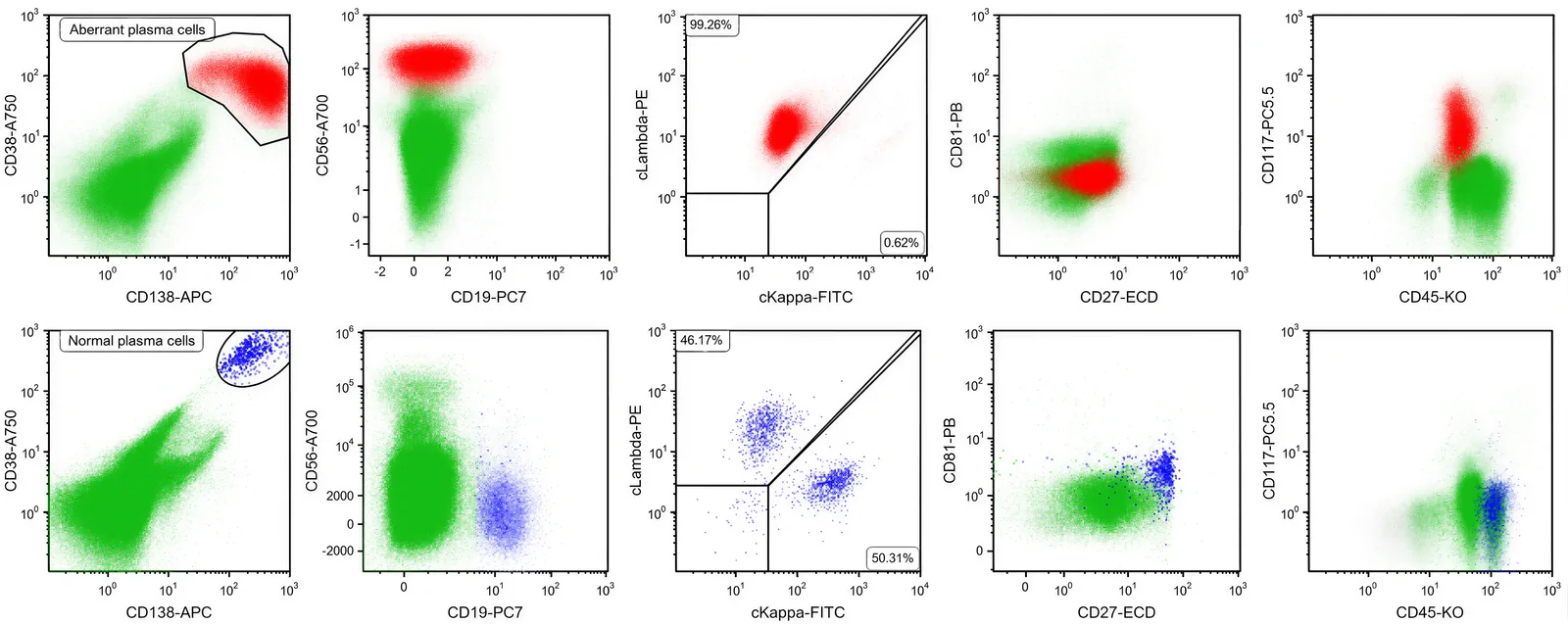
Representative bone marrow immunophenotypic plots used to characterize aberrant clonal plasma cells (red) and residual normal plasma cells (blue); other nucleated cells are shown in green. The panels illustrate CD38/CD138-based plasma-cell identification, aberrant antigen-expression patterns, and cytoplasmic light-chain restriction. cKappa-FITC, cLambda-PE, CD27-ECD, CD117-PC5.5, CD19-PC7, CD138-APC, CD56-A700, CD38-A750, CD81-PB, CD45-KrO.

### 2.3. Grouping by CTPC level

Patients with evaluable peripheral blood CTPC data were stratified according to a previously reported CTPC cutoff of 0.07%.^[6]^ Patients with CTPC levels of ≥0.07% were assigned to the CTPC-higher group, whereas those with CTPC levels of <0.07% were assigned to the CTPC-lower group. Because this study was cross-sectional and did not include survival outcomes, this cutoff was used only as an analytical grouping threshold and not to validate prognostic risk stratification. Because no independent binary diagnostic or clinical outcome was available, a cohort-specific receiver operating characteristic-derived threshold was not estimated.

### 2.4. Clinical and laboratory variables

Baseline clinical and laboratory data were collected at initial diagnosis. These included age, sex, hemoglobin, platelet count, serum calcium, blood urea nitrogen, creatinine, albumin, β2-MG, LDH, IgG, IgA, IgM, serum kappa light chain, and BM plasma cell percentage. Bone marrow involvement was assessed by morphological smear examination and multiparameter flow cytometry. Variables with missing data were analyzed using available cases, and the corresponding denominators were reported where applicable. Variable-specific available-case denominators are provided in Table 1 and the relevant figure legends.

**Table 1:** Baseline characteristics and comparison between CTPC-higher and CTPC-lower groups with false-discovery-rate-adjusted *q* values.

Characteristic	All patients (n = 51)	CTPC-higher (n = 20)	CTPC-lower (n = 21)	*P* value	FDR-adjusted *q* value
Sex (male/female)	28/23	10/10	13/8	.651	0.743
Age, yr	67.00 (59.50–75.00)	73.00 (65.75–77.25)	62.00 (58.00–71.00)	.057	0.101
Hemoglobin, g/L	88.00 (73.50–112.50)	73.50 (65.75–82.50)	107.00 (86.00–115.00)	<.001	0.005
Platelets, ×10^9^/L	127.00 (100.50–182.50)	120.00 (98.50–225.00)	151.00 (101.00–189.00)	.764	0.815
Calcium, mmol/L	2.22 (2.09–2.34)	2.16 (2.05–2.30)	2.23 (2.13–2.35)	.441	0.589
Blood urea nitrogen, mmol/L	6.97 (5.53–11.47)	7.61 (6.11–13.16)	7.08 (5.67–8.95)	.440	0.589
Creatinine, μmol/L	71.50 (59.25–125.00)	81.00 (70.00–140.00)	73.00 (64.00–122.00)	.490	0.603
Albumin, g/L	34.20 (29.10–41.10)	30.90 (25.90–33.83)	38.20 (34.10–42.90)	.006	0.018
β2-microglobulin, mg/L	4.42 (3.00–7.25)	6.97 (4.83–11.95)	3.25 (2.49–5.77)	.004	0.018
LDH, U/L	173.00 (129.00–201.00)	171.00 (124.00–222.00)	166.00 (125.00–195.00)	.837	0.837
IgG, g/L	15.86 (5.20–54.10)	42.34 (7.11–69.97)	7.46 (5.00–21.05)	.031	0.084
IgA, g/L	0.54 (0.25–9.09)	0.32 (0.15–0.93)	0.94 (0.40–9.09)	.091	0.145
IgM, g/L	0.21 (0.15–0.28)	0.20 (0.12–0.21)	0.22 (0.18–0.34)	.044	0.100
Serum kappa light chain, g/L	4.50 (1.27–16.00)	16.00 (2.75–35.50)	2.90 (1.20–8.10)	.051	0.101
BM plasma cells (smear), %	24.50 (9.00–40.00)	34.75 (27.13–61.12)	12.00 (3.50–31.50)	.005	0.018
BM plasma cells (flow), %	6.58 (2.51–17.43)	17.43 (6.07–32.80)	3.75 (1.02–10.22)	.005	0.018

Data are shown as the median (interquartile range). Continuous variables were compared using the Mann–Whitney *U* test, and categorical variables were compared using the chi-square or Fisher exact test.

The corresponding available-case denominators for variables with missing data were: calcium, all n = 49 (higher n = 20; lower n = 21); blood urea nitrogen, all n = 50 (higher n = 19; lower n = 21); creatinine, all n = 50 (higher n = 19; lower n = 21); β2-microglobulin, all n = 44 (higher n = 18; lower n = 18); LDH, all n = 45 (higher n = 19; lower n = 17); IgA, all n = 49 (higher n = 18; lower n = 21); and serum kappa light chain, all n = 48 (higher n = 19; lower n = 21). *q* values were obtained using the Benjamini–Hochberg false-discovery-rate procedure across the Table 1 comparisons.

BM = bone marrow, CTPC = circulating tumor plasma cells, FDR = false discovery rate, Ig = immunoglobulin, LDH = lactate dehydrogenase.

### 2.5. Statistical analysis

Statistical analyses were performed using Python (version 3.11; Python Software Foundation) in the PyCharm integrated development environment (version 2023.3.5). Continuous variables were tested for normality using the Shapiro–Wilk test. Continuous data were summarized as medians with interquartile ranges and compared between the groups using the Mann–Whitney *U* test. Categorical variables are presented as counts and percentages and compared using the chi-square test or Fisher exact test, as appropriate. Spearman rank correlation analysis was used to evaluate the association between CTPC levels and clinical variables. Pairwise correlations among selected variables were visualized using a correlation heatmap. All tests were 2-sided, and *P* < .05 was considered nominally significant. Benjamini–Hochberg false-discovery-rate (FDR) correction was applied separately to the CTPC-higher versus CTPC-lower clinical comparisons, selected CTPC correlations, and surface-antigen MFI comparisons; *q* < 0.05 was considered significant after correction. The supplementary heatmap was descriptive, and its asterisks denote nominal *P* values only. Exploratory binary logistic regression models used CTPC-higher status as the dependent variable and evaluated each selected clinical marker in a separate model adjusted for age and sex. Because of the limited number of CTPC-higher events, predictors were entered in separate parsimonious models; sensitivity models for hemoglobin, albumin, and β2-MG additionally adjusted for BM plasma cell percentage on smear. Results are reported as adjusted odds ratios (ORs) with 95% confidence intervals (CIs). Patients with and without evaluable CTPC data were compared using P values and absolute standardized mean differences to assess potential selection bias.

## 3. Results

### 3.1. Patient characteristics and CTPC group comparison

A total of 51 patients with newly diagnosed MM, including 28 men and 23 women, were included in this study. Among them, 41 patients had evaluable peripheral blood CTPC data and were included in CTPC-related analyses. The median age of these 41 patients was 68.0 years (interquartile range, 60.0–76.0 years). Peripheral blood CTPCs were detected in 34 of the 41 patients, with levels ranging from 0% to 38.32%. Seven patients had undetectable levels of CTPC. According to the predefined 0.07% cutoff, 20 patients were assigned to the CTPC-higher group and 21 to the CTPC-lower group.

The baseline characteristics and intergroup comparisons are shown in Table 1. Compared with the CTPC-lower group, the CTPC-higher group had significantly lower hemoglobin levels (73.5 vs 107.0 g/L; *P* < .001) and albumin levels (30.9 vs 38.2 g/L; *P* = .006). The CTPC-higher group also had higher β2-MG levels (6.97 vs 3.25 mg/L; *P* = .004). Bone marrow plasma cell percentages were significantly higher in the CTPC-higher group in both smear examination (34.75% vs 12.00%; *P* = .005) and flow cytometry (17.43% vs 3.75%; *P* = .005). All 5 comparisons remained significant after FDR correction (*q* ≤ 0.018).

The nominal differences in IgG and IgM levels and the trend in serum kappa light-chain levels did not remain significant after FDR correction. Patients in the CTPC-higher group tended to be older than those in the CTPC-lower group, although the difference was not statistically significant (73.0 vs 62.0 years; *P* = .057). No significant differences were observed in sex distribution, platelet count, serum calcium, blood urea nitrogen, creatinine, LDH, or IgA levels.

Compared with the 10 patients without evaluable peripheral blood CTPC data, the 41 patients included in CTPC analyses had lower hemoglobin levels and higher BM plasma cell percentages on smear examination. These imbalances indicate that selection bias related to CTPC availability cannot be excluded (Table S1, Supplemental Digital Content 1).

### 3.2. Correlation between CTPC levels and BM tumor burden

Peripheral blood CTPC levels were positively correlated with BM plasma cell percentage on smear examination (Spearman ρ = 0.67; *P* < .001; Fig. 2). CTPC levels were also positively correlated with BM plasma cell percentage measured using flow cytometry (ρ = 0.61; *P* < .001). These findings indicate that higher CTPC levels are associated with a greater BM plasma cell burden at diagnosis. Both correlations remained significant after FDR correction (*q* < 0.001).

**Figure 2.**
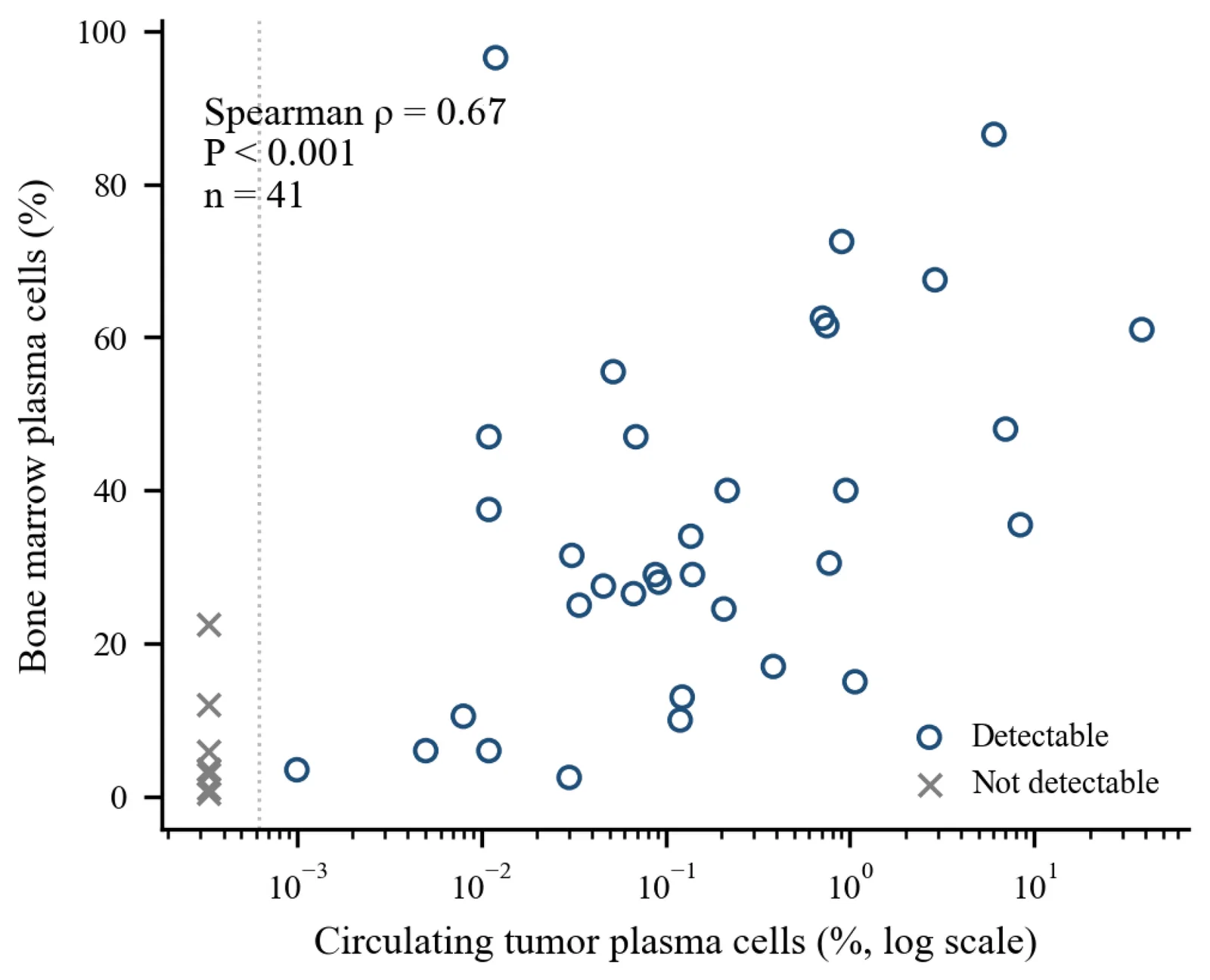
Correlation between circulating tumor plasma cell (CTPC) level and bone marrow plasma cell percentage on smear examination (Spearman ρ = 0.67, *P* < .001, n = 41). The *x*-axis is logarithmic; the 7 patients with 0 recorded CTPC values are displayed at a fixed offset to the left of the dotted separation line for visualization and were retained as zero in the Spearman analysis.

### 3.3. Association between CTPC levels and routine laboratory parameters

CTPC levels were negatively correlated with hemoglobin levels (ρ = −0.68; *P* < .001) and albumin levels (ρ = −0.41; *P* = .009) and positively correlated with β2-MG levels (ρ = 0.46; *P* = .005; Fig. 3). No significant correlations were observed between CTPC and LDH, creatinine, calcium, or IgG levels. The 3 reported correlations remained significant after FDR correction (*q* ≤ 0.017).

**Figure 3.**
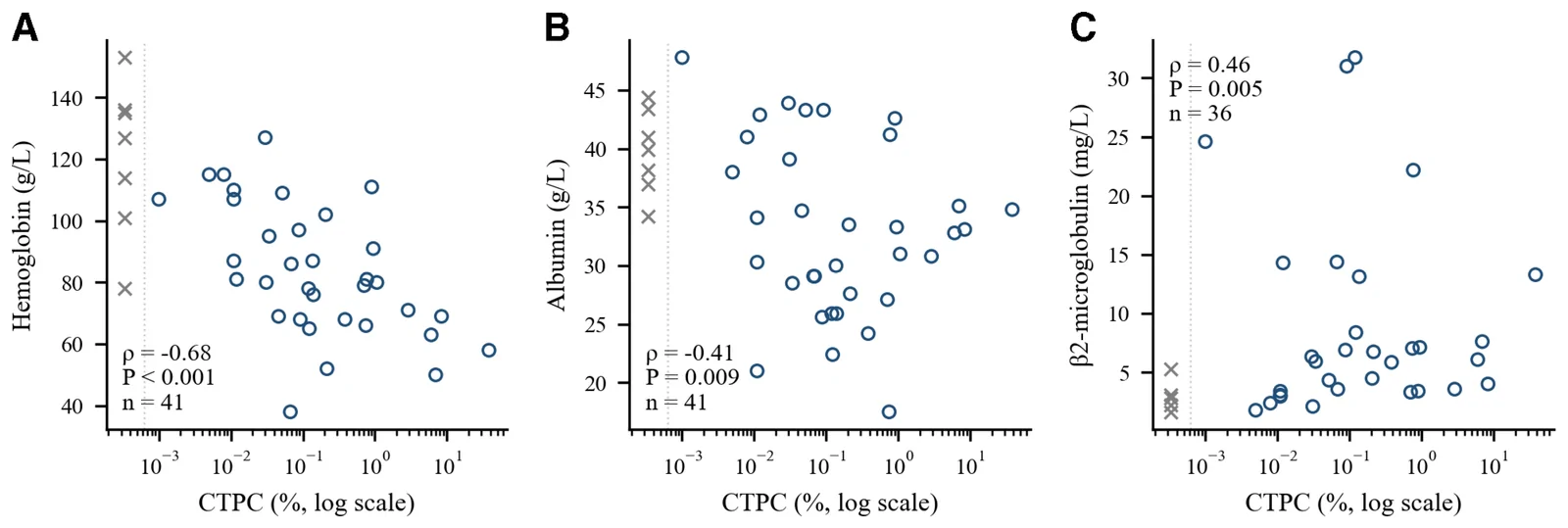
Correlation of CTPC level with (A) hemoglobin (ρ = −0.68, *P* < .001, n = 41), (B) albumin (ρ = −0.41, *P* = .009, n = 41), and (C) β2-microglobulin (ρ = 0.46, *P* = .005, n = 36). The *x*-axis is on a logarithmic scale. The 7 patients with 0 recorded CTPC values are displayed at a fixed offset for visualization and were retained as 0 in the correlation analyses. CTPC = circulating tumor plasma cell.

A Spearman correlation heatmap was used to summarize pairwise associations between CTPC levels and the selected clinical variables (Fig. S1, Supplemental Digital Content 2). The heatmap showed that CTPC levels were mainly associated with BM plasma cell burden, hemoglobin, albumin, and β2-MG, whereas correlations with renal function, calcium metabolism, platelet count, and LDH were not statistically significant.

### 3.4. Group differences in selected clinical parameters

The differences in selected clinical parameters between the CTPC-lower and CTPC-higher groups are shown in Figure 4. Hemoglobin, albumin, β2-MG, and BM plasma cell percentage differed significantly between the groups, consistent with the correlation analyses. All 4 comparisons remained significant after FDR correction (*q* ≤ 0.018).

**Figure 4.**
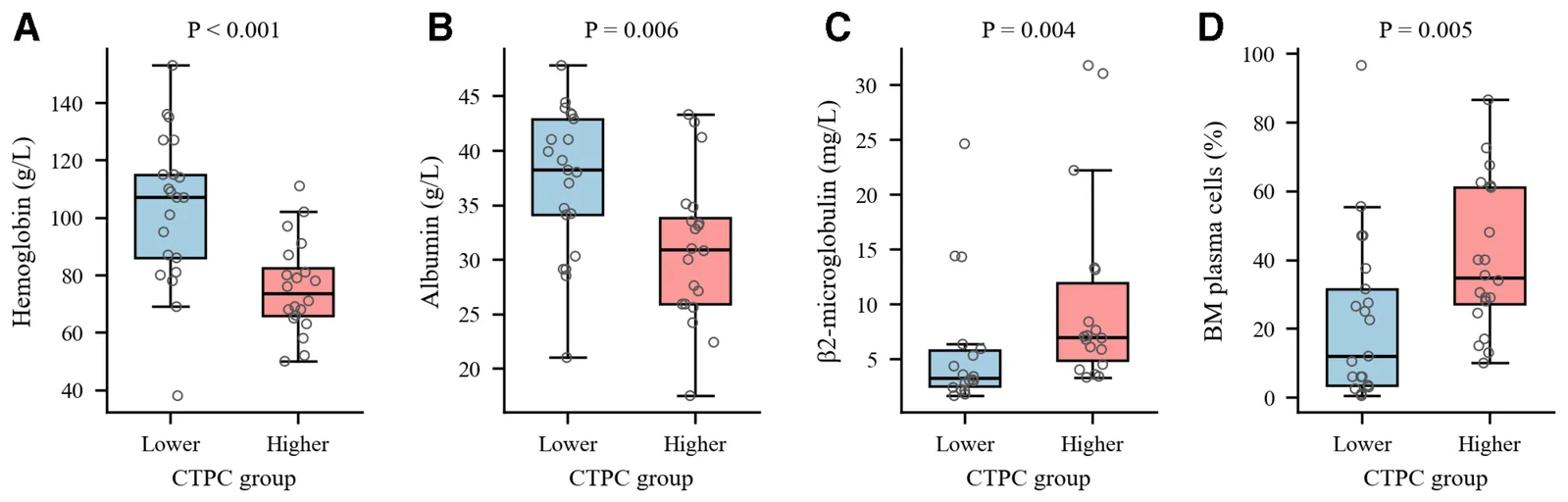
Comparison of (A) hemoglobin (n = 21 vs n = 20), (B) albumin (n = 21 vs n = 20), (C) β2-microglobulin (n = 18 vs n = 18), and (D) bone marrow plasma cell percentage on smear examination (n = 21 vs n = 20) between the CTPC-lower and CTPC-higher groups. *P* values are from the Mann–Whitney *U* test; all 4 comparisons remained significant after false-discovery-rate correction (*q* ≤ 0.018). CTPC = circulating tumor plasma cell.

In exploratory logistic regression adjusted for age and sex, higher hemoglobin (OR per 10 g/L, 0.57; 95% CI, 0.38–0.85; *P* = .006) and albumin (OR per 5 g/L, 0.48; 95% CI, 0.26–0.88; *P* = .018) were associated with lower odds of CTPC-higher status, whereas higher β2-MG (OR per doubling, 2.16; 95% CI, 1.01–4.60; *P* = .046) and BM plasma cell percentage on smear (OR per 10 percentage points, 1.41; 95% CI, 1.01–1.97; *P* = .043) were associated with higher odds. The association for BM plasma cell percentage measured by flow cytometry did not reach statistical significance (OR per 10 percentage points, 1.54; 95% CI, 0.97–2.43; *P* = .068). In sensitivity models additionally adjusted for BM plasma cell percentage on smear, the associations of hemoglobin, albumin, and β2-MG remained nominally significant (all *P* ≤ .041; Table S2, Supplemental Digital Content 3).

### 3.5. Immunophenotypic characteristics of BM plasma cells

The immunophenotypic characteristics of the BM plasma cells from the 51 patients are summarized in Table 2. CD138 was positive in all cases (100.0%), and CD38 was positive or dim in 50 of the 51 cases (98.0%). CD56 was positive in 46 cases and dim in 5 cases, yielding a combined positive/dim rate of 100.0%, whereas CD19 expression was detected in only 1 case (2.0%). CD45 expression was positive or dim in 12 cases (23.5%). Variable expression of CD117 (56.9%) and CD27 (43.1%) was observed. CD81 was positive in 3 cases (5.9%), and no dim cases were recorded. Cytoplasmic light-chain restriction was identified in 50 of the 51 cases, including 29 kappa-restricted and 21 lambda-restricted cases.

Quantitative surface-antigen expression was further compared between the CTPC groups using MFI values (Table 3). CD138 MFI was nominally lower in the CTPC-higher group than in the CTPC-lower group (100.02 vs 176.75; *P* = .043), but no surface-antigen MFI comparison remained significant after FDR correction (all *q* > 0.05).

## 4. Discussion

In this retrospective single-center study of patients with newly diagnosed MM, higher peripheral blood CTPC levels were associated with a greater BM plasma cell burden and more abnormal routine laboratory markers. Patients in the CTPC-higher group had lower hemoglobin and albumin levels, higher β2-MG levels, and higher BM plasma cell percentages. The principal group comparisons and correlations remained significant after FDR correction, and hemoglobin, albumin, β2-MG, and BM plasma cell percentage on smear remained associated with CTPC-higher status in parsimonious age- and sex-adjusted models. These findings are consistent with previous reports linking circulating plasma cells to adverse disease features in MM.^[9–12]^ They extend these observations to a regional clinical cohort assessed using routinely available baseline measures.

The 0.07% CTPC cutoff was adopted from the FORTE trial and used only as an analytical grouping threshold. Other studies have proposed different thresholds, ranging from very low-level CTPC detection to higher values used to identify plasma cell leukemia-like disease.^[13]^ Therefore, this study should not be interpreted as validating a prognostic cutoff. Because no independent binary diagnostic or clinical outcome was available, a cohort-specific receiver operating characteristic-derived cutoff would not have had a valid reference standard.

β2-MG and albumin are components of the International Staging System, and LDH is included in the Revised International Staging System.^[14,15]^ More recent staging approaches also incorporate additional biological variables.^[16]^ In our cohort, CTPC-higher patients had higher β2-MG and lower albumin levels, whereas LDH did not differ significantly. The practical contribution of this study is the integrated assessment of CTPC burden against routinely available markers in a regional real-world cohort, rather than the identification of a new prognostic threshold.

The positive correlation between CTPC levels and BM plasma cell percentage supports a relationship between peripheral blood dissemination and central tumor burden. However, CTPCs should not replace BM evaluation. Prior studies indicate that CTPCs provide information related to, but not identical with, marrow burden,^[17]^ and that serial changes may have clinical relevance.^[18]^ Their role in disease monitoring therefore requires longitudinal validation.

Bone marrow aspiration and biopsy remain essential for MM diagnosis, but sampling variability may limit representation of the entire disease burden. Spatial genomic and transcriptomic heterogeneity in MM supports this concern.^[19,20]^ In this context, peripheral-blood CTPC assessment may provide complementary information regarding systemic dissemination,^[21]^ particularly in regional settings where repeated marrow evaluation or advanced molecular testing may be less accessible.

The BM immunophenotype was broadly consistent with established patterns of clonal plasma cells. The combination of CD38, CD138, CD45, CD19, CD56, and cytoplasmic light-chain restriction is commonly used to distinguish neoplastic from normal or reactive plasma cells.^[22,23]^ The combined CD56 positive/dim rate was 100%, higher than commonly reported. This may reflect the small cohort, local classification thresholds, and inclusion of dim expression in the combined rate; the finding should not be generalized.

Although CD138 has recognized roles in plasma-cell adhesion and dissemination,^[24,25]^ its nominal MFI difference did not remain significant after FDR correction. Because raw MFI was not calibrated to standardized fluorescence units,^[26]^ the MFI analysis is exploratory, and no biological inference is drawn.

This study had several limitations. First, it was retrospective and single-center, with only 41 patients with evaluable peripheral blood CTPC data, limiting statistical power and generalizability. Second, some clinical variables were missing, and patients without evaluable CTPC data differed on several baseline measures; selection bias cannot be excluded. Third, no treatment-response, minimal residual disease, progression-free survival, or overall survival data were available, so prognostic value could not be assessed. Fourth, the 0.07% cutoff was literature-derived rather than validated in this cohort. At the lower acquisition target of 100,000 events, a 0.07% population corresponds to approximately 70 events; precision near the threshold may therefore be limited, and no study-specific limit of detection or lower limit of quantification was established. MFI remained exploratory because raw values were not standardized, and the local protocol was not a formally standardized EuroFlow assay. The multivariable models were intentionally parsimonious because of the small event count, and residual confounding remains possible. Future prospective multicenter studies should evaluate whether standardized CTPC assessment adds independent value and whether longitudinal monitoring improves disease assessment.^[27,28]^

## 5. Conclusion

In this retrospective single-center study, higher peripheral blood CTPC levels were associated with an increased BM plasma cell burden, lower hemoglobin and albumin levels, and higher β2-MG levels in patients with newly diagnosed MM. These associations remained evident in parsimonious age- and sex-adjusted analyses. These findings suggest that CTPC quantification using multiparameter flow cytometry may provide additional information for baseline disease assessment. However, because of the limited sample size, cross-sectional design, and lack of longitudinal outcomes, they do not establish prognostic value. Larger prospective studies are needed to determine whether standardized CTPC assessment adds independent clinical value.

## Author contributions

**Conceptualization:** Heng Tang, Mengyu Zhang.

**Data curation:** Di Hu, Wenqiang Fu, Henggui Hu, Xiaomeng Xu.

**Formal analysis:** Wenwen Chen, Yuehong Li.

**Funding acquisition:** Xiaolei Du.

**Investigation:** Xiaolei Du, Di Hu, Wenqiang Fu, Henggui Hu, Wenwen Chen, Xiaomeng Xu, Yuehong Li.

**Writing – original draft:** Xiaolei Du.

**Writing – review & editing:** Heng Tang, Mengyu Zhang.







## References

[1] Rajkumar SV Dimopoulos MA Palumbo A. International Myeloma Working Group updated criteria for the diagnosis of multiple myeloma. Lancet Oncol. 2014;15:e538–48. 10.1016/S1470-2045(14)70442-5 25439696

[2] Cowan AJ Green DJ Kwok M. Diagnosis and management of multiple myeloma: a review. JAMA. 2022;327:464–77. 10.1001/jama.2022.0003 35103762

[3] John L Poos AM Brobeil A. Resolving the spatial architecture of myeloma and its microenvironment at the single-cell level. Nat Commun. 2023;14:5011. 10.1038/s41467-023-40584-4 PMC10435504 37591845

[4] Vagnoni D Travaglini F Pezzoni V. Circulating plasma cells in newly diagnosed symptomatic multiple myeloma as a possible prognostic marker for patients with standard-risk cytogenetics. Br J Haematol. 2015;170:523–31. 10.1111/bjh.13484 26010293

[5] Tembhare PR Sriram H Khanka T. Circulating tumor plasma cells and peripheral blood measurable residual disease assessment in multiple myeloma patients not planned for upfront transplant. Hemasphere. 2024;8:e63. 10.1002/hem3.63 PMC10983024 38566804

[6] Bertamini L Oliva S Rota-Scalabrini D. High levels of circulating tumor plasma cells as a key hallmark of aggressive disease in transplant-eligible patients with newly diagnosed multiple myeloma. J Clin Oncol. 2022;40:3120–31. 10.1200/JCO.21.01393 35666982

[7] Flores-Montero J de Tute R Paiva B. Immunophenotype of normal vs. myeloma plasma cells: toward antibody panel specifications for MRD detection in multiple myeloma. Cytometry B Clin Cytom. 2016;90:61–72. 10.1002/cyto.b.21265 26100534

[8] Flores-Montero J Sanoja-Flores L Paiva B. Next generation flow for highly sensitive and standardized detection of minimal residual disease in multiple myeloma. Leukemia. 2017;31:2094–103. 10.1038/leu.2017.29 PMC5629369 28104919

[9] Garcés JJ Cedena MT Puig N. Circulating tumor cells for the staging of patients with newly diagnosed transplant-eligible multiple myeloma. J Clin Oncol. 2022;40:3151–61. 10.1200/JCO.21.01365 35666958

[10] Jelinek T Bezdekova R Zihala D. More than 2% of circulating tumor plasma cells defines plasma cell leukemia-like multiple myeloma. J Clin Oncol. 2023;41:1383–92. 10.1200/JCO.22.01226 PMC9995102 36315921

[11] Granell M Calvo X Garcia-Guiñón A. Prognostic impact of circulating plasma cells in patients with multiple myeloma: implications for plasma cell leukemia definition. Haematologica. 2017;102:1099–104. 10.3324/haematol.2016.158303 PMC5451342 28255016

[12] Nowakowski GS Witzig TE Dingli D. Circulating plasma cells detected by flow cytometry as a predictor of survival in 302 patients with newly diagnosed multiple myeloma. Blood. 2005;106:2276–9. 10.1182/blood-2005-05-1858 PMC1895270 15961515

[13] Fernández de Larrea C Kyle R Rosiñol L. Primary plasma cell leukemia: consensus definition by the International Myeloma Working Group according to peripheral blood plasma cell percentage. Blood Cancer J. 2021;11:192. Published 2021 Dec 2. 10.1038/s41408-021-00587-0 PMC8640034 34857730

[14] Rajkumar SV. Multiple myeloma: 2024 update on diagnosis, risk-stratification, and management. Am J Hematol. 2024;99:1802–24. 10.1002/ajh.27422 PMC11404783 38943315

[15] Palumbo A Avet-Loiseau H Oliva S. Revised international staging system for multiple myeloma: a report from International Myeloma Working Group. J Clin Oncol. 2015;33:2863–9. 10.1200/JCO.2015.61.2267 PMC4846284 26240224

[16] D’Agostino M Cairns DA Lahuerta JJ. Second revision of the international staging system (R2-ISS) for overall survival in multiple myeloma: a European myeloma network (EMN) report within the HARMONY project. J Clin Oncol. 2022;40:3406–18. 10.1200/JCO.21.02614 35605179

[17] Gonsalves WI Jevremovic D Nandakumar B. Enhancing the R-ISS classification of newly diagnosed multiple myeloma by quantifying circulating clonal plasma cells. Am J Hematol. 2020;95:310–5. 10.1002/ajh.25709 PMC7724649 31867775

[18] Chakraborty R Muchtar E Kumar SK. Serial measurements of circulating plasma cells before and after induction therapy have an independent prognostic impact in patients with multiple myeloma undergoing upfront autologous transplantation. Haematologica. 2017;102:1439–45. 10.3324/haematol.2017.166629 PMC5541877 28473618

[19] Rasche L Chavan SS Stephens OW. Spatial genomic heterogeneity in multiple myeloma revealed by multi-region sequencing. Nat Commun. 2017;8:268. 10.1038/s41467-017-00296-y PMC5559527 28814763

[20] Merz M Merz AMA Wang J. Deciphering spatial genomic heterogeneity at a single cell resolution in multiple myeloma. Nat Commun. 2022;13:807. 10.1038/s41467-022-28266-z PMC8831582 35145077

[21] Sanoja-Flores L Flores-Montero J Garcés JJ. Next generation flow for minimally-invasive blood characterization of MGUS and multiple myeloma at diagnosis based on circulating tumor plasma cells (CTPC). Blood Cancer J. 2018;8:117. 10.1038/s41408-018-0153-9 PMC6242818 30455467

[22] Leonardos D Benetatos L Apostolidou E. Applications of multiparameter flow cytometry in the diagnosis, prognosis, and monitoring of multiple myeloma patients. Diseases. 2025;13:320. 10.3390/diseases13100320 PMC12563596 41149054

[23] Seegmiller AC. Flow cytometry in the diagnosis and prognosis of multiple myeloma. Semin Diagn Pathol. 2025;42:150942. 10.1016/j.semdp.2025.150942 40743872

[24] Akhmetzyanova I McCarron MJ Parekh S Chesi M Bergsagel PL Fooksman DR. Dynamic CD138 surface expression regulates switch between myeloma growth and dissemination. Leukemia. 2020;34:245–56. 10.1038/s41375-019-0519-4 PMC6923614 31439945

[25] Baert L Manfroi B Quintero M. 3-O sulfation of syndecan-1 mediated by the sulfotransferase HS3ST3a1 enhances myeloma aggressiveness. Matrix Biol. 2023;120:60–75. 10.1016/j.matbio.2023.05.005 37201729

[26] Hofste op Bruinink D Oliva S Rihova L. Standardization of flow cytometric minimal residual disease assessment in international clinical trials – a feasibility study from the European Myeloma Network. Haematologica. 2020;106:1496–9. 10.3324/haematol.2020.267831 PMC8094105 33054135

[27] Garcés JJ San-Miguel JF Paiva B. Biological characterization and clinical relevance of circulating tumor cells: opening the pandora’s box of multiple myeloma. Cancers (Basel). 2022;14:1430. 10.3390/cancers14061430 PMC8946760 35326579

[28] Kumar S Paiva B Anderson KC. International Myeloma Working Group consensus criteria for response and minimal residual disease assessment in multiple myeloma. Lancet Oncol. 2016;17:e328–46. 10.1016/S1470-2045(16)30206-6 27511158

