# Supplementary material for: Circulating tumor plasma cells correlate with tumor burden and routine laboratory markers in newly diagnosed multiple myeloma: A retrospective single-center study
Source: Medicine (Baltimore). 2026 Oct 16;105(42):e51073. doi: 10.1097/MD.0000000000051073 (PMC13680956; doi:10.1097/MD.0000000000051073)
Supplement: Supplementary file 1 [file medi-105-e51073-s001.docx]

**Supplementary Table S1. Comparison of patients with and without evaluable peripheral-blood CTPC data.**

| **Characteristic** | **CTPC evaluable** | **CTPC unevaluable** | **Evaluable n** | **Unevaluable n** | **P value** | **Absolute SMD** |
| --- | --- | --- | --- | --- | --- | --- |
| Sex (male/female) | 23/18 | 5/5 | 41 | 10 | 0.739 | 0.12 |
| Age, years | 68.0 (60.0–76.0) | 65.5 (59.2–68.2) | 41 | 10 | 0.561 | 0.17 |
| Hemoglobin, g/L | 86.0 (69.0–109.0) | 111.5 (90.5–126.8) | 41 | 10 | 0.049 | 0.68 |
| Platelets, ×10^9/L | 131.0 (101.0–192.0) | 123.0 (96.8–134.8) | 41 | 10 | 0.280 | 0.57 |
| Calcium, mmol/L | 2.19 (2.07–2.34) | 2.25 (2.10–2.33) | 41 | 8 | 0.695 | 0.26 |
| Blood urea nitrogen, mmol/L | 7.21 (5.80–11.77) | 5.72 (5.33–7.94) | 40 | 10 | 0.102 | 0.55 |
| Creatinine, μmol/L | 73.0 (66.2–136.0) | 63.5 (55.0–90.8) | 40 | 10 | 0.087 | 0.55 |
| Albumin, g/L | 34.1 (29.1–41.0) | 37.8 (31.8–46.6) | 41 | 10 | 0.280 | 0.41 |
| β2-microglobulin, mg/L | 5.58 (3.26–7.81) | 2.92 (2.70–4.25) | 36 | 8 | 0.070 | 0.74 |
| LDH, U/L | 168.5 (124.5–203.2) | 186.0 (164.0–201.0) | 36 | 9 | 0.250 | 0.10 |
| IgG, g/L | 20.54 (5.43–65.40) | 10.04 (4.15–29.05) | 41 | 10 | 0.313 | 0.25 |
| IgA, g/L | 0.53 (0.23–7.61) | 1.42 (0.48–12.96) | 39 | 10 | 0.244 | 0.04 |
| IgM, g/L | 0.21 (0.14–0.25) | 0.27 (0.21–0.46) | 41 | 10 | 0.042 | 0.71 |
| Serum kappa light chain, g/L | 5.65 (1.57–19.73) | 2.60 (1.23–4.75) | 40 | 8 | 0.166 | 0.47 |
| BM plasma cells (smear), % | 28.00 (10.50–47.00) | 10.50 (6.75–13.12) | 41 | 10 | 0.027 | 0.91 |
| BM plasma cells (flow), % | 9.60 (3.21–20.16) | 3.91 (2.03–6.81) | 41 | 10 | 0.090 | 0.83 |

Data are median (interquartile range) unless otherwise indicated. Continuous variables were compared using the Mann–Whitney U test and sex using Fisher’s exact test. SMD, standardized mean difference; an absolute SMD ≥0.50 was considered a potentially meaningful imbalance. CTPC, circulating tumor plasma cell; BM, bone marrow; LDH, lactate dehydrogenase. P values are nominal; this comparison was descriptive, and absolute SMDs were used to characterize imbalance.
