# Supplementary material for: Circulating tumor plasma cells correlate with tumor burden and routine laboratory markers in newly diagnosed multiple myeloma: A retrospective single-center study
Source: Medicine (Baltimore). 2026 Oct 16;105(42):e51073. doi: 10.1097/MD.0000000000051073 (PMC13680956; doi:10.1097/MD.0000000000051073)
Supplement: Supplementary file 2 [file medi-105-e51073-s003.docx]

**Supplementary Table S2. Exploratory multivariable logistic regression for CTPC-higher status.**

| **Exposure** | **Adjustment** | **n** | **Events** | **Adjusted OR** | **95% CI** | **P value** | **Analysis** |
| --- | --- | --- | --- | --- | --- | --- | --- |
| Hemoglobin per 10 g/L | Age and sex | 41 | 20 | 0.57 | 0.38–0.85 | 0.006 | Primary |
| Albumin per 5 g/L | Age and sex | 41 | 20 | 0.48 | 0.26–0.88 | 0.018 | Primary |
| β2-microglobulin per doubling | Age and sex | 36 | 18 | 2.16 | 1.01–4.60 | 0.046 | Primary |
| BM plasma cells (smear) per 10 percentage points | Age and sex | 41 | 20 | 1.41 | 1.01–1.97 | 0.043 | Primary |
| BM plasma cells (flow) per 10 percentage points | Age and sex | 41 | 20 | 1.54 | 0.97–2.43 | 0.068 | Primary |
| Hemoglobin per 10 g/L | Age, sex, and BM plasma cells (smear) | 41 | 20 | 0.61 | 0.40–0.92 | 0.020 | Sensitivity |
| Albumin per 5 g/L | Age, sex, and BM plasma cells (smear) | 41 | 20 | 0.47 | 0.24–0.91 | 0.024 | Sensitivity |
| β2-microglobulin per doubling | Age, sex, and BM plasma cells (smear) | 36 | 18 | 2.30 | 1.03–5.12 | 0.041 | Sensitivity |

The outcome was CTPC-higher status (CTPC ≥0.07%). Primary models adjusted each exposure separately for age (per 10 years) and sex. Sensitivity models additionally adjusted for bone marrow plasma cell percentage on smear (per 10 percentage points). β2-microglobulin was log2-transformed, so the OR represents each doubling. P values are nominal because the models were exploratory and intentionally parsimonious. OR, odds ratio; CI, confidence interval; CTPC, circulating tumor plasma cell; BM, bone marrow.
